# Associations Between Pulmonary Function and Muscle Strength in Turkish National Karate Athletes

**DOI:** 10.3390/jcm14186370

**Published:** 2025-09-10

**Authors:** Gurkan Tokgoz, Sena Cinarli, Betül Akyol, Caner Aygoren, Aysegul Beykumul, Malte Nejst Larsen, Peter Krustrup, Cíntia França, Élvio Rúbio Gouveia, Fahri Safa Cinarli

**Affiliations:** 1 Faculty of Sport Science, Munzur University, Tunceli 62000, Türkiye; gurkantokgoz@munzur.edu.tr; 2 Faculty of Health Sciences, Kocaeli Health and Technology University, Kocaeli 41301, Türkiye; haticesena.cinarli@kocaelisaglik.edu.tr; 3 Faculty of Sport Science, Inonu University, Malatya 44280, Türkiye; betul.akyol@inonu.edu.tr; 4 Health Sciences Institute, Munzur University, Tunceli 62000, Türkiye; canerkarate4423@gmail.com; 5 Physical Medicine and Rehabilitation Department, Gazi University, Ankara 06560, Türkiye; abeykumul@yahoo.com; 6 Department of Sports Science and Clinical Biomechanics, University of Southern Denmark, DK-5230 Odense, Denmark; mnlarsen@health.sdu.dk (M.N.L.); pkrustrup@health.sdu.dk (P.K.); 7 Danish Institute for Advanced Study (DIAS), University of Southern Denmark, DK-5230 Odense, Denmark; 8 Department of Physical Education and Sport, University of Madeira, 9020-105 Funchal, Portugal; cintia.franca@staff.uma.pt (C.F.); erubiog@staff.uma.pt (É.R.G.); 9 Laboratory for Robotics and Engineering Systems, Interactive Technologies Institute, 9020-105 Funchal, Portugal; 10 Center for the Interdisciplinary Study of Gerontology and Vulnerability, University of Geneva, 1227 Carouge, Switzerland; 11 Interdisciplinary Centre for the Study of Human Performance, Faculty of Human Kinetics, University of Lisbon, 1495-751 Lisbon, Portugal

**Keywords:** pulmonary function, muscle strength, karate, martial arts

## Abstract

**Background**: Respiratory efficiency is considered important in karate due to its role in sustaining muscular performance during high-intensity actions. This study examined the association between pulmonary function and isometric muscle strength in national-level karate athletes. **Methods**: A total of 23 elite karate athletes (mean age: 23.0 ± 2.3 (mean ± SD) years) participated in the study. Pulmonary function was assessed using a digital spirometer, while isometric handgrip, lower back, and leg strength were measured using a dynamometer. The correlation between pulmonary function and isometric strength was analyzed, and linear regression was employed to examine the predictive capacity of pulmonary parameters for muscle strength. **Results**: The results revealed significant correlations, ranging from large to very large, between pulmonary function and isometric muscle strength, with correlation coefficients from 0.639 to 0.812 (*p* < 0.01). Pulmonary function was strongly associated with isometric strength, accounting for 27% to 67% of the variance (*p* < 0.05). Multiple regression analysis revealed that pulmonary function parameters accounted for 71% of the variance in handgrip strength, 47% in leg strength, and 71% in back strength (*p* < 0.05). **Conclusions**: These findings highlight the strong associations between pulmonary function and isometric muscle strength in elite karate athletes. The results emphasize the importance of pulmonary health and respiratory muscle function in athletic performance, particularly for sports requiring high-intensity, dynamic movements like karate. Future longitudinal studies are needed to explore the mechanisms underlying the association and potential implications, and for training and performance optimization.

## 1. Introduction

Karate is a combat sport that places substantial demands on both the respiratory and muscular systems through high-intensity actions and complex motor skills [1]. While kumite primarily relies on aerobic energy pathways due to continuous dynamic movements, kata depends more heavily on anaerobic alactic systems, particularly the ATP-phosphocreatine pathway [2]. Energy system contributions during kumite have been estimated at approximately 77.8% aerobic, 16.0% anaerobic alactic, and 6.2% anaerobic lactic metabolism [3]. Simulated karate bouts can elicit energy expenditures of ~16 kcal/min and heart rates peaking at 187 bpm [4], underscoring the sport’s reliance on optimal cardiopulmonary function.

Efficient pulmonary function is critical for meeting these physiological demands by supporting oxygen delivery, carbon dioxide clearance, and overall respiratory efficiency during high-intensity efforts [5,6]. Athletic performance and cardiopulmonary fitness are fundamentally dependent on pulmonary function and effective lung ventilation [7]. Breathing mechanics during high-intensity exercise are intricately linked to respiratory muscle fatigue, which can influence overall performance [8]. Pulmonary function plays an important role in regulating oxygen delivery to working muscles through its effects on vasomotor outflow, vascular conductance, and blood flow distribution [9]. Increased pulmonary demand during strenuous activity may compromise limb blood flow and oxygen transport, contributing to muscle fatigue and limiting athletic capacity [10,11]. These mechanisms emphasize the importance of respiratory muscle function in mitigating fatigue and sustaining performance, particularly in sports requiring prolonged isometric and dynamic muscle contractions. Although pulmonary function has been studied only to a limited extent in karate athletes [12], emerging evidence suggests its potential role in competitive success. Supporting this notion, elite karatekas with higher pulmonary parameters, such as forced vital capacity and forced expiratory volume in one second, have been shown to achieve superior outcomes in international competitions [13].

Beyond its direct respiratory role, well-developed pulmonary function contributes to muscular strength by enhancing oxygen transport, improving gas exchange, and delaying respiratory muscle fatigue, indirectly supporting force production during high-intensity exertion [14]. These effects are proposed to result from mechanisms such as the modulation of blood flow distribution and the maintenance of trunk stability through respiratory muscle activity. A central element of this link is the generation of intra-abdominal pressure (IAP), whereby diaphragmatic contraction increases abdominal pressure, stiffens the spine, and provides core stability to facilitate efficient force transfer. Moreover, adequate pulmonary function supports IAP and postural control, both essential for effective force generation during isometric tasks [15,16]. The relevance of IAP extends beyond athletic performance; impaired IAP regulation has been associated with low back pain, reduced trunk stability, and increased fall risk in older adults [17,18]. Thus, elite karate athletes offer a valuable physiological model to investigate how respiratory mechanics and IAP generation contribute to muscular strength under conditions of maximal demand. The importance of muscular strength is further underscored by evidence from elite kumite karatekas, where maximal strength has been shown to be closely associated with sprinting and jumping performance, highlighting its relevance for explosive and functional athletic tasks [19].

Despite these plausible links, most existing research has focused on clinical populations or older adults [20], with limited attention directed toward athletic cohorts [21]. This gap restricts our understanding of how pulmonary parameters influence muscular strength in high-performance karate athletes. To date, no study has specifically investigated the relationship between pulmonary function and muscle strength in elite karate competitors. Therefore, the present study aims to elucidate the association between pulmonary function and isometric muscle strength in national-level karate athletes. Pulmonary function parameters are hypothesized to exhibit strong associations with isometric strength performance.

## 2. Materials and Methods

### 2.1. Study Design

This cross-sectional study examined the relationship between pulmonary muscle function and isometric muscle strength in national-level karate athletes. To ensure reliable measurements, participants were instructed to refrain from exercising for at least 72 h before testing and to maintain their usual dietary habits. A certified trainer administered strength tests, while an experienced physiotherapist conducted pulmonary function assessments. The study protocol was approved by the University Ethics Committee (protocol number: 2024/5488). All procedures were performed in accordance with the ethical standards of the Declaration of Helsinki, and written informed consent was obtained from all participants.

### 2.2. Participants

Sample size calculations were performed using G*Power software (version 3.1.9.3; Heinrich Heine University Düsseldorf, Germany), based on a prior study evaluating the association between handgrip strength and forced vital capacity in elite athletes (r^2^ = 0.38) [22]. With a statistical power of 80% (1 − β = 0.80), a minimum of 18 participants was required. To account for potential dropouts, 23 national-level karate athletes were recruited (9 males, mean age 24.2 ± 2.2 years; 14 females, mean age 22.3 ± 2.2 years). All participants were national athletes: 8 competed in kata (including one individual world champion and three world champions in team events) and 15 competed in kumite (including one world champion, one world silver medalist, and one world bronze medalist). None of the participants had a history of health conditions that could impair their ability to perform the breathing or strength tests. Inclusion criteria were age between 18 and 25 years, participation in international competitions within the last two years, and the ability to fulfill the requirements of the breathing and strength tests. Participants with obstructive lung disease were excluded.

### 2.3. Body Composition

Anthropometric measurements were conducted following the protocols of the International Society for the Advancement of Kinanthropometry, with the technical error of measurement maintained below 1% [23]. Height was measured using a portable stadiometer to the nearest 0.1 cm (Seca Ltd., Bonn, Germany), and body mass was determined using a digital weighing scale (Model 813, Seca Ltd., Hamburg, Germany).

### 2.4. Isometric Handgrip Strength Test

Handgrip strength (HGS) was assessed using a digital dynamometer (Takei 5401, Takei Scientific Instruments Co., Ltd., Tokyo, Japan) with participants in a standing position, elbow flexed at 90°, forearm in a neutral position, and wrist extended between 0° and 30° [24]. Following a demonstration and a familiarization trial, participants performed three maximal efforts, each lasting 6 s, with 30 s rest intervals between trials. The highest recorded value was accepted as the maximum HGS. The dominant hand, defined as the writing hand, was evaluated. The intra-class correlation coefficient (ICC) for HGS measurement was 0.98, indicating excellent reliability.

### 2.5. Isometric Leg and Back Strength Test

Leg and back strength were measured using a hydraulic dynamometer (Baseline, Fabrication Enterprises Inc. New York, NY, USA). For leg strength assessment, participants adopted a semi-squat position with the knees flexed at 135°, verified using a goniometer positioned 10 cm above the patella. Participants were instructed to extend their legs with maximal effort. The highest value from three trials, separated by 30 s rest intervals, was recorded as the peak leg strength. For back strength assessment, participants kept their legs straight and bent forward until their index fingers reached the kneecaps. They then fully extended their arms to grip the dynamometer rod [25]. The intra-class correlation coefficients (ICC) for both leg and back strength measurements exceeded 0.90, indicating good reliability.

### 2.6. Pulmonary Function Test

Pulmonary function was assessed according to the American Thoracic Society and European Respiratory Society standards. Procedures followed the updated 2019 technical standards for spirometry [26] and interpretive strategies were aligned with the most recent 2022 ATS/ERS technical standard [27]. Spirometry was performed using a COSMED Pony FX spirometer (COSMED srl, Rome, Italy), calibrated in accordance with the manufacturer’s guidelines. Participants were seated with hips and knees flexed at 90° and wore a nose clip throughout the procedure. Spirometry parameters included forced vital capacity (FVC), forced expiratory volume in one second (FEV_1_), peak expiratory flow (PEF), and maximal voluntary ventilation (MVV). For the MVV test, participants were instructed to breathe as rapidly and deeply as possible for 12 s while seated and wearing a nose clip. The maneuver was repeated at least twice, and the highest reproducible value was extrapolated to one minute and used for analysis, in accordance with ATS/ERS standards. Tests were repeated until the two highest values for FEV_1_ or FVC were within 0.15 L of each other, and the highest value was used for analysis.

### 2.7. Statistical Analysis

All statistical analyses were conducted using IBM SPSS software (version 24; IBM Corporation, Armonk, NY, USA). Normality was assessed with the Shapiro–Wilk test, given the sample size (<50). Pearson’s correlation coefficients and regression analyses were used to evaluate the strength of association and variance explained between pulmonary function and isometric strength parameters. Correlation strength was interpreted as follows: r = 0–0.30, small; 0.31–0.49, moderate; 0.50–0.69, large; 0.70–0.89, very large; and 0.90–1.00, nearly perfect. The independent parameter was entered into stepwise multiple linear regression analyses when significant correlations were identified. Multicollinearity was assessed using the variance inflation factor (VIF) and tolerance, with VIF >10 and tolerance <0.1 indicating multicollinearity. Results are presented as mean ± standard deviation (SD) and 95% confidence intervals (CIs). Statistical significance was set at *p* < 0.05.

## 3. Results

The demographic characteristics, training status, pulmonary muscle function parameters, and isometric strength measures are presented in Table 1 and Table 2. All variables were normally distributed. Values are expressed as mean ± standard deviation (SD) with 95% confidence intervals (CI).

**Figure 1:**
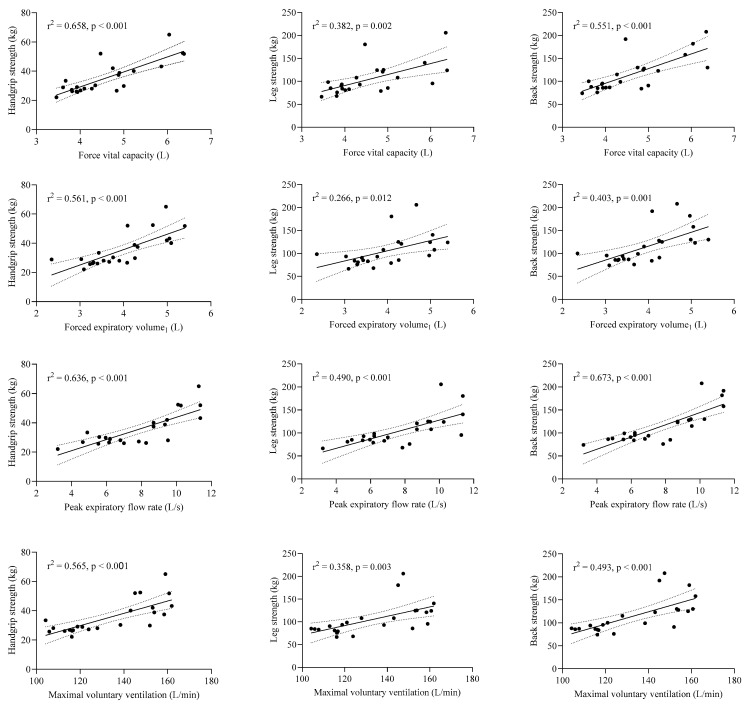
Illustrates the correlation between muscle strength and pulmonary function. Individual data points are presented, as well as a linear regression line with 95% CI.

Figure 1 illustrates the relationships between handgrip strength and pulmonary function parameters, revealing very large positive correlations and statistically significant simple linear regression results for forced vital capacity (FVC, L) (r = 0.811, *p* < 0.001), forced expiratory volume in one second (FEV_1_, L) (r = 0.749, *p* < 0.001), peak expiratory flow (PEF, L/s) (r = 0.798, *p* < 0.001), and maximum voluntary ventilation (MVV, L/min) (r = 0.752, *p* < 0.001).

Similarly, large to very large positive correlations were observed between leg strength and pulmonary function parameters: FVC (r = 0.618, *p* = 0.002), FEV_1_ (r = 0.516, *p* = 0.012), PEF (r = 0.700, *p* < 0.001), and MVV (r = 0.598, *p* = 0.003). In addition, back strength exhibited large to very large positive correlations with pulmonary function measures: FVC (r = 0.742, *p* < 0.001), FEV_1_ (r = 0.635, *p* = 0.001), PEF (r = 0.820, *p* < 0.001), and MVV (r = 0.702, *p* < 0.001). Furthermore, pulmonary function parameters were strong predictors of isometric strength, explaining 56–66% of the variance in handgrip strength (r^2^ = 0.56–0.66), up to 49% in leg strength (r^2^ = 0.27–0.49), and as much as 67% in back strength (r^2^ = 0.40–0.67).

Significant associations were identified between pulmonary function parameters and isometric muscle strength across all tests. The models for handgrip strength (F = 14.390, *p* < 0.001, adjusted r^2^ = 0.709), leg strength (F = 5.869, *p* = 0.003, adjusted r^2^ = 0.470), and back strength (F = 14.746, *p* < 0.001, adjusted r^2^ = 0.714) were all statistically significant, with pulmonary function collectively accounting for 70.9%, 47.0%, and 71.4% of the variance in these outcomes, respectively (Table 3). Collinearity diagnostics (tolerance and VIF values) are presented in Table 4, confirming that multicollinearity was not a concern (all VIF < 10 and tolerance > 0.1).

## 4. Discussion

This study demonstrated significant positive correlations between pulmonary function and isometric muscle strength in national-level karate athletes. Pulmonary function showed strong associations with isometric strength outcomes. These findings underscore the contribution of pulmonary function to overall muscular performance, with consistent associations observed across strength outcomes.

Pulmonary function varies across sports in response to discipline-specific demands, yet consistently exceeds that of sedentary individuals, establishing it as an important indicator of athletic adaptation. A study reported that boxers and taekwondo athletes exhibit superior vital capacity, forced vital capacity, and forced expiratory volume compared to sedentary controls, reflecting sport-specific respiratory adaptations [28]. Respiratory muscle strength also differs among combat sports; judo and Muay Thai athletes display higher inspiratory pressures than boxers and taekwondo athletes, likely due to differences in static and dynamic loading patterns [29]. While our study did not compare athletes by performance level, previous research in junior elite kumite karatekas demonstrated that higher pulmonary function parameters (e.g., FVC, FEV_1_, MVV) are associated with greater competitive success [13]. This highlights the potential role of pulmonary efficiency in karate performance, aligning with our findings on its association with isometric strength. To the best of our knowledge, no prior study has specifically examined pulmonary function in tier 4 national-level karate athletes, underscoring the novelty of our investigation.

Handgrip strength is a critical attribute in athletic disciplines that require explosive power and grip control, such as throwing, punching, clinching, martial arts, rowing, and swinging [30,31,32]. Moreover, previous studies have shown that handgrip strength serves as a reliable predictor of success in martial arts, underscoring its relevance as a performance metric [33]. In the present study, handgrip strength showed the strongest associations with pulmonary function, explaining a greater proportion of variance than lower limb strength. This finding is consistent with evidence suggesting that upper limb muscle strength has a more direct influence on ventilation efficiency and, consequently, physical performance and its relationship with pulmonary function [16]. Supporting this, a significant association between upper limb strength and pulmonary function (adjusted r^2^ = 0.255, *p* < 0.01) was reported [34]. Collectively, these results highlight the dual role of upper limb muscles in contributing to both mechanical performance and respiratory efficiency.

Conversely, the weaker correlations observed between pulmonary function and lower body strength may reflect the differing functional roles of these muscle groups concerning respiration. The mechanics of breathing primarily involve the diaphragm and intercostal muscles, along with accessory muscles such as those of the upper limbs, particularly during intense physical exertion or fatigue [35]. In contrast, lower limb muscles are mainly responsible for locomotion and power generation, with limited direct involvement in pulmonary mechanics. In a study of healthy older adults, a significant relationship was found between handgrip strength and pulmonary function, but not between knee extension torque and pulmonary function (*p* > 0.05) [20]. However, research in athletes from other sports, including judo and rowing, suggests that knee extensor and flexor strength can still be significantly associated with pulmonary function, with explained variances ranging from 44% to 66% [21]. Collectively, these findings indicate that while the relationship between pulmonary function and muscle strength is evident across different muscle groups, it may vary according to the anatomical and functional roles of the muscles involved.

It is important to recognize that anthropometric variables such as body mass and stature may serve as significant confounders in the observed relationships between pulmonary function and muscle strength. Taller and heavier individuals inherently possess greater absolute lung volumes and muscle strength, a phenomenon well documented across both human physiology and sports science literature [36]. The necessity of normalization in functional performance measures is emphasized, particularly using allometric scaling methods—for example, scaling force by body mass to the exponent of ~0.67—so as to diminish body-size-related confounding effects [37]. In pulmonary research, the associations between height and both FVC and FEV_1_ have been consistently reported, underscoring the value of adjusting for stature when interpreting spirometric outcomes [38].

In response to this critical consideration, we conducted exploratory analyses using normalized variables—namely, strength adjusted for body mass and pulmonary functions normalized to height. As anticipated, the correlations were somewhat attenuated but generally remained within the moderate to large range. In contrast, partial correlations controlling for body mass and stature markedly reduced the strength of the associations, and most were no longer statistically significant. This pattern suggests that body size partly mediates the observed relationships and may account for a larger proportion of the shared variance than initially assumed. Given these findings, future studies with larger sample sizes should implement rigorous normalization techniques and potentially allometric scaling to better isolate the independent contribution of respiratory and muscular capacities. The detailed results of these exploratory analyses are provided in Supplementary Table S1.

The findings of this study have important implications for training and performance optimization in karate athletes. Given the significant relationship observed between pulmonary function and isometric strength, particularly in the upper limb and back muscles, integrating respiratory muscle training into conditioning programs could enhance both pulmonary efficiency and muscular performance. Strengthening the respiratory muscles has been shown to mitigate exercise-induced respiratory muscle fatigue, improve oxygen delivery, and reduce limb fatigue during sustained activity. Future research should explore the effects of targeted respiratory muscle training on strength and performance outcomes in elite martial artists.

Although this study did not specifically examine injury risk, the interplay between pulmonary function and isometric strength may have implications beyond performance, particularly in injury prevention. Pulmonary function and isometric strength maintain intra-abdominal pressure, dynamic postural stability, and neuromuscular control during high-intensity movements [39,40]. These factors are recognized as protective against musculoskeletal overload and compensatory movement patterns that could predispose athletes to injury, especially in sports such as karate, where rapid transitions between dynamic and static actions are required. This conceptual link underscores the potential value of incorporating respiratory and isometric strength assessments into comprehensive injury prevention frameworks in combat sports.

This study has several limitations that warrant consideration. The cross-sectional design precludes causal inferences regarding the relationship between pulmonary function and muscle strength; longitudinal studies are needed to evaluate the effects of training on these parameters over time. Furthermore, only isometric strength was assessed in the present study. While isometric tests were chosen due to their high reliability and widespread use in sports science research, karate performance predominantly relies on dynamic and explosive strength, such as rapid force production during punches, kicks, and jumps. The hypothesized link through IAP may, in theory, exert an even stronger effect during explosive and dynamic actions than in static conditions. Thus, although our findings demonstrated significant associations between pulmonary function and isometric strength, these relationships might underestimate the potential contribution of IAP to sport-specific performance. Incorporating assessments of isotonic and isokinetic contractions, as well as sport-specific dynamic performance measures (e.g., jump height, punch/kick velocity), could provide a more comprehensive understanding of the interaction between pulmonary function, IAP, and muscular strength in karate athletes. Given the relatively small sample size and the number of correlations conducted, the potential for type I error cannot be excluded. The absence of formal correction for multiple testing represents a limitation, and future studies with larger samples should apply appropriate adjustment procedures. Despite these limitations, the current study advances knowledge of the physiological mechanisms underlying performance in elite karate. The findings provide a foundation for the future integration of pulmonary and muscular training strategies to optimize performance in martial arts.

## 5. Conclusions

This study demonstrated significant correlations between isometric muscle strength and pulmonary function in national-level karate athletes, with handgrip strength exhibiting the strongest association with pulmonary function. The findings demonstrate strong associations between respiratory efficiency and upper limb strength, suggesting a potential link to overall athletic performance. The results suggest that pulmonary function and IAP mechanisms may contribute to force production and trunk stability, which are physiologically relevant in karate. Future research should employ longitudinal and sport-specific designs to further clarify the relationship between pulmonary function, IAP, and muscular strength in combat sports.

## Figures and Tables

**Table 1:** Descriptive statistics for the participant characteristics in karate athletes (*n* = 23).

Variables	Mean ± Standard Deviation	95% CI
Age (years)	23.0 ± 2.3	22.0, 24.1
Height (cm)	170.0 ± 7.7	166.9, 173.6
Weight (kg)	65.0 ± 11.0	60.2, 69.8
Body mass index (kg/m^2^)	22.2 ± 2.1	21.3, 23.1
Training experience (years)	9.2 ± 2.2	8.3, 10.2
Training frequency (session/week)	4.6 ± 0.5	4.4, 4.8
Training frequency (hours/week)	6.9 ± 0.7	6.0, 7.5

**Table 2:** Descriptive statistics of the participants’ pulmonary muscle functions and isometric muscle strength values (*n* = 23).

Variables	Mean ± Standard Deviation	95% CI
Handgrip strength (kg)	35.3 ± 11.2	30.4, 40.1
Leg strength (kg)	105.1 ± 34.3	90.2, 119.9
Back strength (kg)	114.6 ± 38.0	98.2, 131.1
FVC (L)	4.59 ± 0.88	4.20, 4.97
FEV_1_ (L)	3.96 ± 0.80	3.61, 4.30
PEF (L/s)	7.76 ± 2.34	6.74, 8.77
MVV (L/min)	133.0 ± 20.0	124.4, 141.7

FVC = forced vital capacity; FEV_1_ = forced expiratory volume in the first second; PEF = peak expiratory flow rate; MVV = maximal voluntary ventilation.

**Table 3:** Multiple regression analyses of the prediction of isometric strength values from pulmonary functions.

Dependent Variables	Independent Variables	*β*	*t*	*r* ^2^	*r* ^2^_adj_	F	*p*-Value
Handgrip strength (kg)	FVC	−0.526	1.977	0.762	0.709	14.390	<0.001
	FEV_1_	−0.124	−0.448				
	PEF	0.437	2.429				
	MVV	0.106	0.473				
Leg strength (kg)	FVC	0.535	1.490	0.566	0.470	5.869	0.003
	FEV_1_	−0.516	−1.377				
	PEF	0.572	2.352				
	MVV	0.169	0.559				
Back strength (kg)	FVC	0.612	2.323	0.766	0.714	14.746	<0.001
	FEV_1_	−0.491	−1.785				
	PEF	0.643	3.601				
	MVV	0.137	0.618				

FVC = forced vital capacity; FEV_1_ = forced expiratory volume in the first second; PEF = peak expiratory flow rate; MVV = maximal voluntary ventilation.

**Table 4:** Collinearity diagnostics for independent variables.

Variables	Tolerance	Variance Inflation Factor
FVC (L)	0.187	5.342
FEV_1_ (L)	0.172	5.823
PEF (L/s)	0.408	2.451
MVV (L/min)	0.263	3.798

## Data Availability

The data that support the findings of this study are available from the corresponding author upon reasonable request.

## Supplementary Materials

The following supporting information can be downloaded at: https://www.mdpi.com/article/10.3390/jcm14186370/s1, Table S1: Normalized correlations and partial correlations between strength and pulmonary function indices.

## Abbreviations

The following abbreviations are used in this manuscript:

FVC	forced vital capacity
FEV_1_	forced expiratory volume in the first second
IAP	intra-abdominal pressure
PEF	peak expiratory flow rate
MVV	maximal voluntary ventilation
