# Supplementary material for: Associations Between Pulmonary Function and Muscle Strength in Turkish National Karate Athletes
Source: J Clin Med. 2025 Sep 10;14(18):6370. doi: 10.3390/jcm14186370 (PMC12470806; doi:10.3390/jcm14186370)
Supplement: Supplementary file 1 [file jcm-14-06370-s001.zip › jcm-3781071-supplementary.pdf]

Supplementary Table S1. Normalized correlations and partial correlations between strength and pulmonary function indices

Variable	FVC	FEV_1	PEF	MVV
Handgrip strength	r = 0.737**<br>pr = 0.527*	r = 0.660**<br>pr = 0.413	r = 0.512*<br>pr = 0.317	r = 0.580**<br>pr = 0.142
Leg strength	r = 0.604**<br>pr = 0.384	r = 0.470*<br>pr = 0.189	r = 0.560**<br>pr = 0.334	r = 0.534**<br>pr = 0.198
Back strength	r = 0.746**<br>pr = 0.441*	r = 0.594**<br>pr = 0.200	r = 0.651**<br>pr = 0.365	r = 0.610**<br>pr = 0.048

*FVC = forced vital capacity; FEV1 = forced expiratory volume in the first second; PEF = peak expiratory flow rate; MVV = maximal voluntary ventilation. r = Pearson correlation using normalized variables (strength adjusted for body mass, pulmonary indices normalized by height); pr = partial correlation coefficient controlling for body mass and height. * $p < 0.05$, ** $p < 0.01$*
